# 739. Impact of Alcohol-based Nasal Decolonization’s Method of Documentation in Reducing Methicillin-resistant *Staphylococcus aureus* Hospital-associated Infections

**DOI:** 10.1093/ofid/ofad500.800

**Published:** 2023-11-27

**Authors:** Shawn Alderman, Crystal Heishman, Richard Skiff

**Affiliations:** UofL Health - Jewish Hospital, Heart Hospital, Louisville, Kentucky; UofL Health, Louisville, Kentucky; UofL Health, Louisville, Kentucky

## Abstract

**Background:**

Infections related to multi-drug resistant organisms (MDRO) are increasingly difficult to treat and contribute to elevated mortality, prolonged length of stay and financial burden. At a 382-bed academic medical center, universal decolonization using an alcohol-based nasal decolonizing agent is performed twice daily on all inpatients, as well as chlorhexidine gluconate (CHG) bathing on select populations. Numerous studies cite decolonization as an effective reduction method for MDRO such as methicillin-resistant *Staphylococcus aureus* (MRSA). Unfortunately, documentation method is a seldomly considered variable.

The purpose of this review was to evaluate the impact of documentation method for an alcohol-based nasal decolonization agent in reducing MRSA hospital-associated infections.

**Methods:**

The study included all National Healthcare Safety Network (NHSN) defined hospital associated laboratory identified (Lab ID) MRSA blood infections from July 2019 through March 2023. Data was divided into descriptive categories with number of infections noted. The baseline period included population specific decolonization (n=35), followed by universal decolonization documentation via nursing task list (n=13), and concluded with electronic medication administration record (EMAR) implementation (n=7). The Kruskal Wallace Test was utilized for evaluation due to non-normative distribution.

**Results:**

A Kruskal Wallace H Test indicated a statistical significance in medians between the task list and EMAR H(1) =5.10, *p=0.024*. There was no significance noted between the baseline and task list H(1 =0.04), *p=0.841*. Medians between baseline and EMAR approached significance but did not meet for this study H(1) =3.24, *p=0.072*.

Clinically, there was a reduction in MRSA Lab ID of 48% between baseline and task list, followed by a 46% reduction between task list and EMAR. Overall reduction from baseline to EMAR was 80%.

**Conclusion:**

Reduction of infection is crucial for patient safety and quality outcomes. Documentation is not typically highlighted as a risk reduction method. This study noted a significant decrease in hospital-associated MRSA Lab ID events after transition of documentation from the nursing task list to the EMAR.

**Disclosures:**

**All Authors**: No reported disclosures

